# Climate Change Sensitivity and Regional Differences of the Upper Limit of Montane Deciduous Broad‐Leaved Forests Across the Northern Hemisphere

**DOI:** 10.1002/ece3.73561

**Published:** 2026-05-11

**Authors:** Youheng Li, Fang Han, Chuanrong Li, Kun Li, Xiaoyong Li, Yan Lv, Xiaolong Xu, Junxin Zhao, Ziqiang Lei

**Affiliations:** ^1^ School of Civil Engineering and Geomatics Shandong University of Technology Zibo China; ^2^ Mountain Tai Forest Ecosystem Research Station of State Forestry Administration Forestry College of Shandong Agricultural University Tai'an China

**Keywords:** climate change, cloud model, northern hemisphere, regional differences, sensitivity index, upper limit of montane deciduous broad‐leaved forests

## Abstract

Montane deciduous broad‐leaved forests across the Northern Hemisphere serve as sensitive ecological indicators of climate change, yet the climate change drivers of the upper limit of montane deciduous broad‐leaved forests (ULMDBs) remain difficult to quantify. Here, we introduce a novel cloud model‐based analytical framework that integrates multisource remote sensing data to extract ULMDB locations and associated impact factors. Using the digital features of the weight coefficient cloud model, we derive a temperature sensitivity index (TSI), a precipitation sensitivity index (PSI), and a comprehensive sensitivity index (CSI), enabling a quantitative assessment of hemispheric‐scale differences in ULMDB climate change responses. Our results reveal pronounced regional differences. In humid regions, 70% of mountains show TSI contribution rates exceeding 50%, indicating temperature‐dominated controls. In arid and semi‐arid regions, more than 80% of mountains exhibit PSI contribution rates above 50%, demonstrating strong sensitivity to precipitation. ULMDBs in East Asian study region show notably higher climate change sensitivity, with a mean CSI of 9.909 and a distinct “increase–decrease” latitudinal pattern, whereas ULMDBs in European study region show a monotonic latitudinal decline. Most ULMDBs occur within humid continental climates (68% of the sample), where 58% show TSI contribution rates above 50%, reflecting greater sensitivity to temperature than to precipitation. Sensitivity indices further suggest that potential responses are most likely where TSI is high, followed by areas with high CSI, whereas PSI‐dominated regions show weaker upward potential. The widespread regional differences indicate that ULMDB climate change sensitivity is governed not by single factors but by the interactions among temperature, precipitation, and regional geographic conditions. The proposed cloud model framework provides a transferable approach for quantifying ecological boundary sensitivity under uncertainty and offers new tools and perspectives for understanding climate change responses of montane forest ecotones and other climate‐sensitive transition zones.

## Graphical abstract

We developed a cloud model–based framework integrating multisource remote sensing data to quantify the climate change sensitivity of the upper limit of montane deciduous broad‐leaved forests (ULMDBs) across the Northern Hemisphere. Results reveal strong regional differences, with temperature dominating ULMDB sensitivity in humid regions and precipitation exerting greater control in arid and semi‐arid regions, leading to distinct regional patterns. This framework offers a transferable approach for assessing ecological boundary responses to climate change under uncertainty.

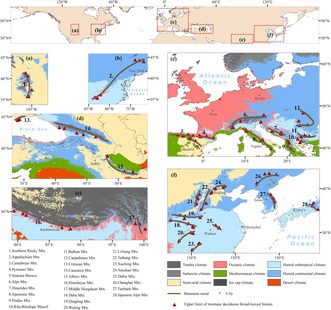

## 1 Introduction

According to the 2023 IPCC Synthesis Report (*IPCC* 2023), global temperatures from 2001 to 2020 increased by 0.99°C relative to 1850–1900, with the rate of global surface temperature rise since 1970 being the highest for any 50‐year period in the past 2000 years. Mountains are among the most fragile environments on Earth (Tse‐Ring et al. 2010), and changes in montane vertical zonation serve as critical indicators of montane ecosystem dynamics (Sun and Cheng 2014).

The timberline, a key boundary within vertical zonation (Li and Tao 1989), is recognized as an ecologically fragile zone and an early warning indicator of global change, making it an ideal subject for climate change monitoring (Barnes 1991; Mu et al. 2023; Ogle and Reynolds 2004; Wolfe 1979; Wang et al. 2024). Climate warming has been widely reported to influence timberline dynamics in transition zones, which have already triggered and are expected to exacerbate ecological issues (Dhar 2012; Griesbauer and Bevington 2024). Under ongoing climate change, timberline dynamics and their driving factors exhibit significant spatial heterogeneity and scale dependence (Yan et al. 2023). However, the mechanisms underlying timberline responses to climate change remain insufficiently understood. Local‐scale studies on timberline distribution may obscure global drivers (Körner 2007), and large‐scale comparative analyses across regions are often constrained by observational limitations and data availability. Although some studies have employed machine learning to identify the impacts of climate factors on timberline location and predict its responses to climate change using climate scenario data and Shared Socioeconomic Pathways (SSPs) (Mkrtchian and Mueller 2025), model reliability is often constrained by parameter selection and challenges in validation at larger scales.

Global climate change is reshaping timberlines, with climatic controls exhibiting regional differences. While some researchers have developed new sensitivity indices to quantify the impact of individual climate factors on timberlines (Gottfried et al. 2012; Zeng et al. 2022), these studies often overlook the complex interactions among different climate factors and are typically limited to smaller spatial scales (Yu et al. 2015; Tse‐Ring et al. 2010; Wieser 2012). At larger scales, due to significant differences in climatic conditions, vegetation responses to climate factors display pronounced spatial heterogeneity, leading to substantial regional differences in timberline sensitivity to climate change (Fang et al. 2005; Ogle and Reynolds 2004). Therefore, quantitatively comparing and analyzing timberline sensitivity to climate change across larger scales and diverse regions, as well as delineating climate‐driven regional differences, is critical to understanding timberline response mechanisms and represents a significant yet understudied issue in current research.

At local and mountain scales, timberline distribution exhibits both fuzziness and randomness as a result of multiple interacting environmental and climatic factors. While each factor contributes to timberline distribution, none acts as a sole determinant, leading to inherent uncertainty in their effects, resulting in uncertainty in their impacts. To address the pervasive uncertainty in timberline studies, researchers have developed the distributed height cloud model to reveal the fuzziness and randomness of timberline distribution (Mu et al. 2023). Additionally, the weight coefficient cloud model has been established to quantify the spatial heterogeneity and scale dependence of impact factors on timberlines (Wang et al. 2024). Existing studies suggest that the digital features of the weight coefficient cloud model for the upper limit of montane deciduous broad‐leaved forests (ULMDBs) can reflect its sensitivity to climate change (Körner 2007; Yan et al. 2023).

Climate is a key factor shaping major ecological communities worldwide (Tanaka 1998). As climate change intensifies, it profoundly impacts forest ecosystems by affecting tree growth, mortality, and reproduction processes (Yoshino 1978). Warm‐temperate forests are particularly sensitive to climate change and precipitation regime shifts, and these forests are often distributed in densely populated, economically developed regions, making them highly vulnerable to climate change impacts (Xie and Cheng 1994). Deciduous broad‐leaved forests, as typical vegetation in high‐altitude temperate and subtropical regions of the Northern Hemisphere (Barnes 1991; Liu et al. 2010), are widely distributed in humid and semi‐humid regions (Wolfe 1979). These forests generally exhibit high species diversity and play a crucial role in maintaining the structural stability and functional integrity of regional ecosystems (Meng et al. 2025; Gao et al. 2021). Their complex community structure enables them to contribute significantly to biodiversity conservation and ecosystem regulation (Meng et al. 2025) while also providing essential ecosystem services, including carbon cycling (Chen et al. 2025), hydrological regulation (Bakker et al. 2019), and regional ecological security (Wei et al. 2024). The formation and evolution of deciduous broad‐leaved forests are strongly regulated by climatic factors, particularly long‐term hydrothermal conditions, and their spatial distribution reflects, to some extent, the process of climatic evolution, providing important insights into forest ecosystem dynamics (Meng et al. 2025; Li et al. 2025). Previous studies have shown that the formation of forest types and the differentiation of leaf phenology (e.g., evergreen versus deciduous) are fundamentally driven by regional climatic conditions, especially the long‐term evolution of monsoon systems (Meng et al. 2025). Under this context, due to their wide distribution and pronounced climatic heterogeneity, deciduous broad‐leaved forests exhibit significant spatial variability in their sensitivity to climate change (Li, Manzanedo, et al. 2023), with the effects of climate change being particularly amplified at ULMDBs. Therefore, understanding the potential responses of ULMDBs from a climate sensitivity perspective is critical for interpreting changes in warm‐temperate ecosystems of the Northern Hemisphere under ongoing climate change.

Given the high sensitivity to climate change and pronounced regional response differences of montane deciduous broad‐leaved forests in the Northern Hemisphere under global warming, clarifying the sensitivity of ULMDBs to various climate factors is essential for understanding the climate adaptation mechanisms of montane forest ecosystems. However, at the hemispheric scale, a unified analytical framework and quantitative method for characterizing the sensitivity of ULMDBs to multiple climatic factors, as well as for identifying their regional differences and dominant driving mechanisms, remain lacking. To address this gap, this study integrates Google Earth imagery, high‐resolution land cover, DEM, and climate datasets to extract impact factors and distribution locations of ULMDBs across 28 mountains in the Northern Hemisphere. On the basis of these data, we developed a distributed height cloud model and a weight coefficient cloud model at the mountain scale for ULMDBs. Using the digital features of the weight coefficient cloud model, we propose single‐factor and comprehensive sensitivity indices for ULMDB sensitivity to climate change, validated using NDVI change rates at corresponding spatial locations. This approach enables quantitative identification and regional differentiation of ULMDB sensitivity to climate change. Within this framework, the term “sensitivity to climate change” is primarily used to characterize the potential sensitivity of ULMDBs to variations in climatic factors, rather than to directly observe or attribute temporal shifts in forest boundary positions. By overcoming the limitations of single‐factor sensitivity analyses, this method effectively addresses the challenges of quantifying uncertainty and fuzziness in traditional approaches, providing a new tool for exploring climate‐driven ecological response mechanisms across regional scales.

## 2 Materials and Methods

### 2.1 Study Area

**FIGURE 1:**
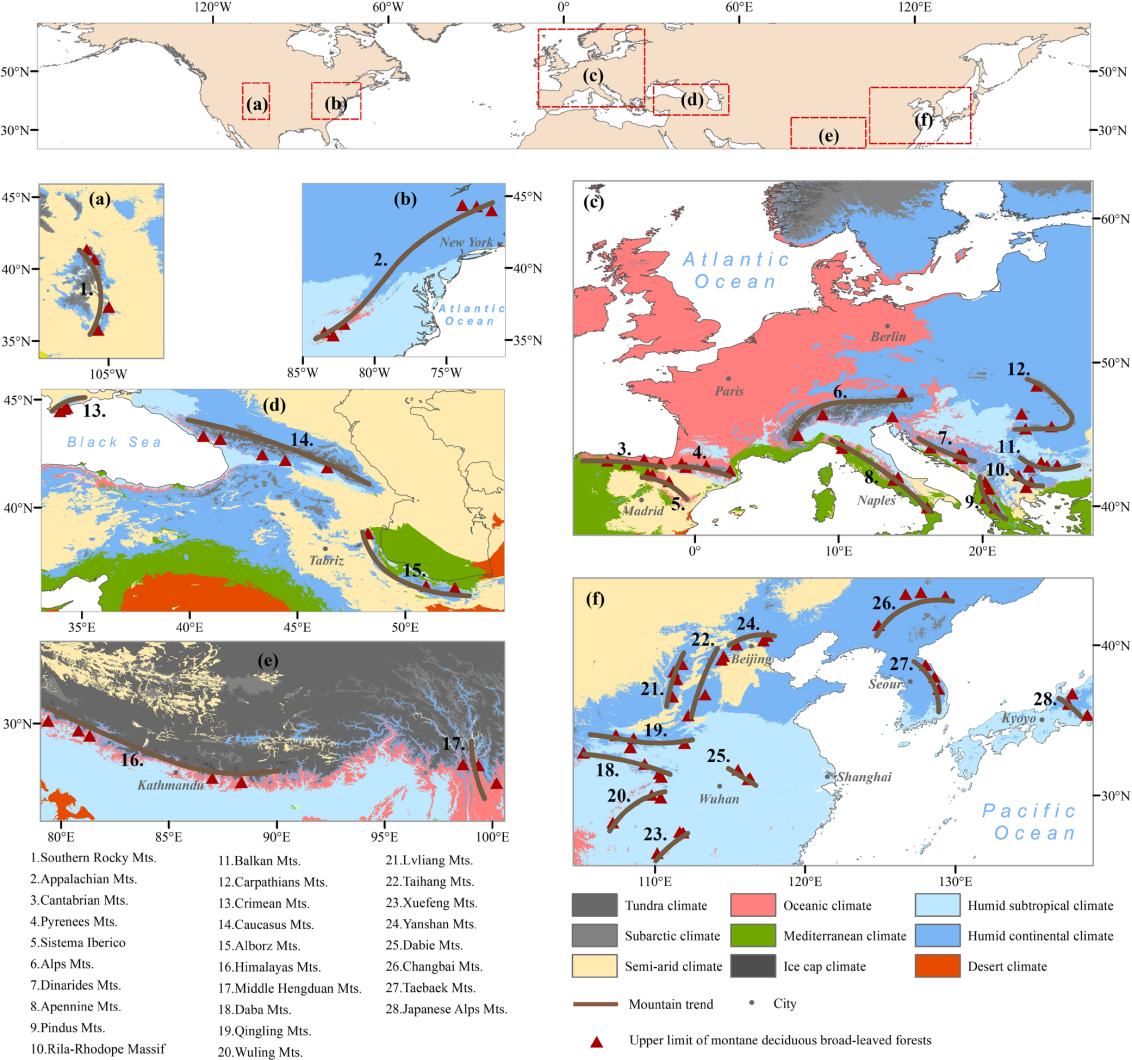
Distribution of ULMDB locations in the Northern Hemisphere. Regional climate types were classified according to the 1991–2020 Köppen climate classification (Beck et al. 2023).

This study compiled literature records of ULMDBs from 28 representative mountains in the Northern Hemisphere (see Supporting Information), including the Alps, Himalayas, and Appalachians (Figure 1). These mountains, characterized by diverse geological histories, climate types, and ecosystems, serve as ideal regions for studying the climate change sensitivity of ULMDBs. The altitudinal range of ULMDBs in these areas spans 700–3600 m, with latitudes ranging from 27° N to 47° N, covering a geographical extent across Eurasia and North America. The study areas have an annual mean precipitation of approximately 750–1500 mm and an annual mean temperature of around 10°C. High‐resolution Google Earth remote sensing imagery and land cover data were used to precisely locate the ULMDB distribution in these regions.

The European study region includes Southern Europe, Southwestern Europe, the Alpine region, and the Black Sea coastal areas. In this region, deciduous broad‐leaved forests are predominantly dominated by European beech ( *Fagus sylvatica* ) (Jahed et al. 2020), often mixed with species of *Quercus* spp. and *Castanea* spp. At mid to lower elevations, these forests typically form *Fagus sylvatica* forests, whereas in Southeastern Europe and along the Black Sea coast, *Fagus orientalis* becomes the dominant species. With increasing elevation, these forests gradually transition into coniferous forests dominated by *Picea abies* and *Abies alba* , which form the timberline. In the Carpathian Mountains, pure beech forests dominated by *Fagus sylvatica* are widely distributed (Hobi et al. 2015). In parts of the Apennine Mountains, *Fagus sylvatica* can extend upward to the timberline and become the dominant species (Bonanomi et al. 2020).

The East Asian study region includes eastern China, the Korean Peninsula, and the Japanese archipelago. Deciduous broad‐leaved forests are mainly composed of *Quercus* spp., *Acer* spp., and *Betula* spp., forming typical temperate deciduous broad‐leaved forests, with upper distribution limits generally higher than those in Europe. In the Qinling Mountains, the deciduous broad‐leaved forest zone is most extensive, with an upper limit of approximately 2800 m (Zhang et al. 2022). Timberlines in this region are also primarily formed by coniferous species such as fir and pine, although in some areas they are composed of deciduous broad‐leaved species; for example, in Changbai Mountain, the timberline is dominated by *Betula ermanii* (Dong et al. 2025).

Other study regions in the Northern Hemisphere are more scattered, mainly located in North America, Central Asia, and South Asia, and exhibit considerable variation in forest types. The Appalachian Mountains are characterized by Northern hardwood forests dominated by *Fagus* spp., *Acer* spp., and *Betula* spp. (Cogbill and White 1991). In contrast, the Alborz Mountains are dominated by *Fagus orientalis* forests, accompanied by *Carpinus betulus* , *Acer velutinum*, and *Quercus macranthera* (Jahed et al. 2020).

### 2.2 Data Source

The Digital Elevation Model (DEM) data were obtained from the NASA SRTM V3 product, with a spatial resolution of 30 m (NASA JPL 2013). Mountain boundary data were sourced from the GMBA Mountain Inventory v2 (Snethlage et al. 2022a, 2022b). Land cover type data were obtained from the Global 30 m fine land cover product in 2020 (Liu and Zhang 2020). Global monthly mean temperature data were obtained from the Yangtze River Middle Reaches Geoscientific Data Center, with a spatial resolution of 1000 m and a temporal range from 2001 to 2010. The global 30 arc‐second precipitation dataset was derived from the CHELSA 2001–2010 monthly mean data and used to calculate annual mean precipitation (Karger et al. 2017). NDVI data were obtained from NASA's MODIS MOD13A2 product, with a spatial resolution of 1000 m and a temporal range from 2001 to 2015 (Didan 2021). Google Earth imagery derived from Landsat 7, with a spatial resolution of 30 m, was used for the period 2001–2010.

### 2.3 Extraction of ULMDBs

Based on literature records, this study identified and verified 107 ULMDB locations across 28 mountains in the Northern Hemisphere using Google Earth. For each location, the deciduous broad‐leaved forest and coniferous forest classes were extracted from the land cover data. Considering that, within montane altitudinal zonation, the deciduous broad‐leaved forest belt is typically located below the coniferous forest belt, only the upper boundary of the deciduous broad‐leaved forest class underlying the coniferous forest class was identified as the ULMDB position. This identification was further refined through visual interpretation using multi‐temporal Google Earth imagery.

To ensure accuracy, the three‐dimensional perspective in Google Earth was adjusted during interpretation, and a minimum ground distance of 400 m between adjacent location points was maintained to avoid multiple points falling within the same raster cell. In total, 4021 ULMDB location points were obtained, with their longitude, latitude, and elevation recorded.

It should be noted that the identification of ULMDBs was not based on the distribution of individual tree species, but on the elevational range of forest belts documented in the literature, in combination with the deciduous broad‐leaved forest class derived from land cover data. In studies where only dominant species information was available, such information was incorporated as an auxiliary reference only when the reported species functioned as constructive species and formed a stable deciduous broad‐leaved forest belt. However, species information itself was not used as a direct criterion for ULMDB delineation.

Based on the global aridity index calculated by Trabucco (Trabucco and Zomer 2019) and the aridity classification scheme proposed by UNEP (UNEP. Governing Council 1997), regions with an aridity index below 0.5 were classified as arid and semi‐arid regions, while others were designated as humid regions. Subsequently, specific climate types for ULMDB locations were further delineated using the Köppen climate classification.

### 2.4 Extraction of Impact Factors

Five impact factors were selected: aspect (Fankhauser 1901; He et al. 2015), terrain relief (Chen et al. 2013), mountaintop effect (Cairns 2013), average temperature in January (Körner 1998; Shen et al. 2017), and average annual precipitation (Wang et al. 2017). For each location point, ArcMap software was used to extract its impact factors. The selection basis and calculation method of the impact factors are shown in (Table 1).

**TABLE 1:** Instructions for calculation of impact factors.

Impact factors	Calculation method	Selection basis
Aspect	Reclassified into four classes: 1 (Shady slope), 2 (Semi‐shady slope), 3 (Sunny slope), and 4 (Semi‐sunny slope)	Determines solar radiation exposure, influencing photosynthesis and microclimate.
Terrain relief	$P=H_{\max}-H_{\min}$ $H_{\max}=$ maximum elevation in the window $H_{\min}=$ minimum elevation in the window	Reflects topographic variability, associated with soil erosion, water retention, and habitat complexity.
Mountaintop effect	Surface area of the mountain above the ULMDBs	The existence of isolated peaks and their vertical boundaries will limit plant growth and renewal.
Average temperature in January	2001–2010 average.	Low temperature conditions will limit plant growth and renewal.
Average annual precipitation	2001–2010 average.	Precipitation is considered a key factor affecting timberline distribution.

### 2.5 Construction of the Distributed Height Cloud Model and the Weight Coefficient Cloud Model

Using location points in each ULMDB locations as analysis units, a multiple linear regression model was established in SPSS 26, with the ULMDB distribution height y as the dependent variable and the impact factors (*x*_1_, *x*_2_, …, *xᵢ*) as independent variables, to construct the regression equation:

(1) $$y=a_{1}x_{1}+a_{2}x_{2}+a_{3}x_{3}+\ldots +a_{i}x_{i}+b$$

where y represents the ULMDB distributed height; $x_{i}$ denotes the *i*‐th impact factor; $a_{i}$ is the regression coefficient for the *i*‐th factor; and b is the constant term.

Assuming the total influence of the i impact factors on ULMDB distribution height is 100%, the relative contribution of each factor to the ULMDB distribution height was calculated based on the magnitude of standardized regression coefficients, yielding the weight coefficient vector for ULMDB distribution height impact factors: $A_{1}={\left[a_{1},a_{2},a_{3},\ldots ,a_{i}\right]}^{T}$. This process was repeated for m analysis units to obtain the weight coefficient matrix: $A=\left[A_{1},A_{2},A_{3},\ldots ,A_{m}\right]$.

Following the cloud model concept proposed by Deyi Li, the distributed height cloud model and the weight coefficient cloud model for ULMDBs were constructed. In the distributed height cloud model, three digital features—expectation (Ex), entropy (En), and hyperentropy (He)—were used to characterize the overall properties of ULMDB distribution height. Ex represents the expectation of ULMDB distribution height; En quantifies the range of variation in ULMDB distribution height, reflecting the uncertainty in the distribution height range, with larger En indicating a more macroscopic concept and greater fuzziness and randomness; He measures the uncertainty of En, representing the randomness of sample occurrences in the qualitative concept and reflecting the variability in ULMDB distribution height, with larger He indicating less concentrated height distribution.

Using MATLAB, the digital features were calculated with the backward cloud generator algorithm, and cloud diagrams were generated using the forward cloud generator. Additionally, mathematical models were established with Ex and En as dependent variables and longitude and latitude as independent variables to explore their spatial distribution patterns.

In the weight coefficient cloud model, Ex, En, and He were used to characterize the overall features of impact factors weight coefficients. Ex represents the expectation of the impact factors' weights; En quantifies the range of variation in impact factors' weights, reflecting the uncertainty in their influence on ULMDB distribution height; He indicates the spatial heterogeneity of impact factors' weights, with smaller He suggesting less spatial variation and more stable influence on ULMDB distribution height.

Based on the weight coefficient matrix, the digital features of the weight coefficient cloud model were calculated using the backward cloud generator algorithm, and cloud model diagrams were generated using the forward cloud generator algorithm, yielding the weight coefficient cloud model for ULMDB impact factors as follows:

(2) $$\begin{array}{c} A=\left[A_{1},A_{2},A_{3},\ldots ,A_{m}\right]=\left[\begin{array}{ccc} {\mathrm{Ex}}_{1} & {\mathrm{En}}_{1} & {\mathrm{He}}_{1}\\ {\mathrm{Ex}}_{2} & {\mathrm{En}}_{2} & {\mathrm{He}}_{2}\\ {\mathrm{Ex}}_{3} & {\mathrm{En}}_{3} & {\mathrm{He}}_{3}\\ \vdots & \vdots & \vdots \\ {\mathrm{Ex}}_{i} & {\mathrm{En}}_{i} & {\mathrm{He}}_{i} \end{array}\right] \end{array}$$

where ${\mathrm{Ex}}_{i}$, ${\mathrm{En}}_{i}$ and ${\mathrm{He}}_{i}$ are the expectation, entropy, and hyperentropy of the *i*‐th impact factor's weight, and *m* is the total number of impact factors.

### 2.6 Construction of Climate Change Sensitivity Indices

For climate‐related factors (e.g., temperature and precipitation), a high Ex with low En and He in the weight coefficient cloud model indicates that the ULMDB distribution height is highly sensitive to that factor. The single‐factor sensitivity index for ULMDB sensitivity to climate change was established as follows:

(3) $$\begin{array}{c} {\mathrm{SI}}_{i}=\frac{E_{x}}{E_{n}+H_{e}}\times \frac{N_{i}}{N} \end{array}$$

where ${\mathrm{SI}}_{i}$ is the single‐factor sensitivity index of ULMDB distribution height to a given climate factor in the *i*‐th analytical unit $E_{x}$, $E_{n}$ and $H_{e}$ are the digital features of the factor's weight, $N_{i}$ is the number of location points in the *i*‐th unit, and 𝑁 is the total number of location points of ULMDB locations.

Based on single‐factor indices, the comprehensive sensitivity index was constructed by summing the contribution rates of all m factors:

(4) $$\begin{array}{c} {\mathrm{CSI}}_{i}=\left(\frac{E_{x1}}{E_{n1}+H_{e1}}+\frac{E_{x2}}{E_{n2}+H_{e2}}+\cdots +\frac{E_{\mathrm{xm}}}{E_{\mathrm{nm}}+H_{\mathrm{em}}}\right)\times \frac{N_{i}}{N} \end{array}$$

where ${\mathrm{CSI}}_{i}$ is the comprehensive sensitivity index of ULMDBs to climate change for the *i*‐th unit. This index accounts for errors due to differences in the number of location points, enabling comparisons of ULMDB sensitivity to climate change across different scales, regions, and types.

The contribution rate of the single‐factor sensitivity index to the comprehensive sensitivity index, $P_{i}$ was calculated as:

(5) $$\begin{array}{c} P_{i}=\frac{{\mathrm{SI}}_{i}}{{\mathrm{CSI}}_{i}} \end{array}$$

If the $P_{i}$ exceeds 50%, it indicates that the ULMDB at that mountain is dominated by the corresponding climatic factor.

It should be noted that the sensitivity indices developed in this study are designed to characterize the response intensity of ULMDBs to variations in climatic factors. The results therefore reflect potential response characteristics rather than actual boundary shift processes.

### 2.7 Validation of the Sensitivity Indices

Using NDVI data from the past 15 years at location points, the interannual NDVI change rate was calculated via simple linear regression:

(6) $$\begin{array}{c} {\text{NDVI}}_{\text{slopej}}=\frac{n\times \sum_{i=1}^{n}i\times {\text{NDVI}}_{i}-\sum_{i=1}^{n}i\sum_{i=1}^{n}{\text{NDVI}}_{i}}{n\times \sum_{i=1}^{n}i^{2}-{\left(n\times \sum_{i=1}^{n}i\right)}^{2}} \end{array}$$

where ${\text{NDVI}}_{\text{slopej}}$ is the NDVI change rate from 2001–2015 for the *j*‐th location point, and ${\text{NDVI}}_{i}$ is the NDVI value in year *i*.

The mean absolute value of NDVI change rates at location points was calculated, representing the overall NDVI response to climate change in the analysis unit. A larger value indicates a stronger NDVI response to climate change.

Using SPSS 26, the Spearman correlation coefficient between the sensitivity index and the NDVI response to climate change was calculated.

## 3 Results

### 3.1 Spatial Distribution Characteristics of ULMDBs in the Northern Hemisphere

**FIGURE 2:**
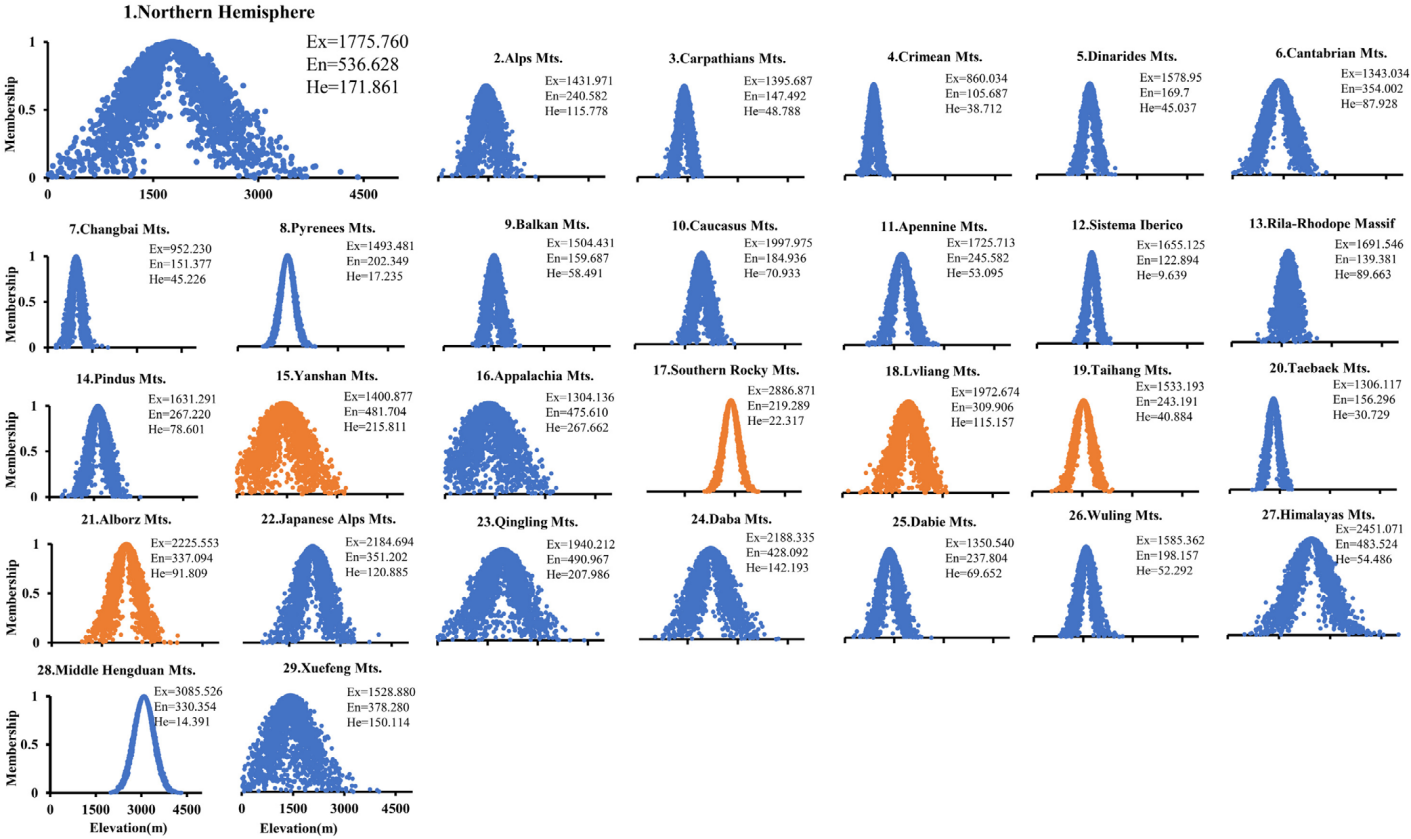
Distributed height cloud model of ULMDBs across the Northern Hemisphere. (1) Overall cloud model representing ULMDB distribution heights across 28 mountains. (2–29) Individual cloud models for each mountain. Blue cloud droplets indicate samples from humid regions, while yellow droplets represent samples from arid and semi‐arid regions, arranged from high to low latitude. The x‐axis represents cloud droplets, and the y‐axis indicates the degree of membership (0–1), reflecting the extent to which each droplet belongs to the expectation (Ex).

ULMDBs in the Northern Hemisphere are primarily concentrated in the East Asian and European study region, accounting for 79% of the total sample (Figure 1). The digital features of the distributed height cloud model for ULMDBs across the Northern Hemisphere (Figure 2) indicate an Ex of 1775.760 m, an En of 536.628 m, and a He of 171.861 m, suggesting significant variability in ULMDB distribution height.

**FIGURE 3:**
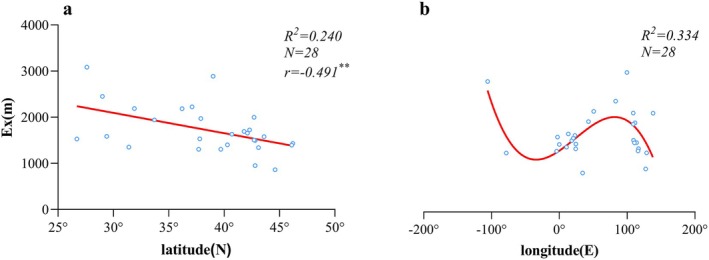
Relationship between the centroid of ULMDB height distribution (Ex) and latitude/longitude across 28 mountains in the Northern Hemisphere. (a, b) show the relationships between Ex and latitude and longitude, respectively. r denotes the correlation coefficient; ** indicates significance at ρ < 0.01; R^2^ represents the coefficient of determination; N is the sample size. Red solid lines indicate the fitted regression lines. The geographic coordinates of each ULMDB were calculated as the mean latitude and longitude of its location points.

The relationship between the Ex of the ULMDB height distribution and geographic coordinates (Figure 3) shows a negative correlation with latitude, consistent with findings of Tan Jing (Tan 2009) on timberline elevation variations across Eurasia. Ex exhibits a fluctuating trend with longitude, with maximum values observed in the Southern Rocky Mts. in central western North America (2886.871 m) and the Middle Hengduan Mts. of the western Qinghai‐Tibet Plateau (3085.526 m), indicating that ULMDB distribution height generally increases from coastal areas toward inland regions.

ULMDBs are predominantly distributed in humid continental climate zones, comprising 68% of the total sample. In mountains with sufficient elevation, ULMDBs tend to cluster at the boundary between humid continental and subarctic climate zones.

In humid regions, the maximum Ex for ULMDBs (3085.526 m) occurs in the Middle Hengduan Mts., while the minimum (860.034 m) is found in the Crimean Mts. The maximum He (267.662 m) is observed in the Appalachian Mts., and the minimum (14.391 m) in the Middle Hengduan Mts. In arid and semi‐arid regions, ULMDBs are typically scattered in inland areas or at the margins of monsoon zones, likely due to climate conditions such as drought and water scarcity. In these regions, the maximum Ex (2886.871 m) is found in the Southern Rocky Mts., and the minimum (1400.877 m) in the Yanshan Mts. The maximum He (215.811 m) occurs in the Yanshan Mts., and the minimum (22.237 m) in the Southern Rocky Mts.

### 3.2 Spatial Heterogeneity of ULMDB Impact Factors in the Northern Hemisphere

The average contribution rates of temperature, mountaintop effect, and precipitation to ULMDB distribution height across the 28 mountains are 47%, 20%, and 24%, respectively. Temperature has a significantly higher contribution rate than other factors, making it the primary driver of ULMDBs reaching their upper limit, followed by the mountaintop effect. Terrain relief and aspect contribute only 5% and 4%, respectively.

Among the 28 mountains, the mountaintop effect exceeds a 20% contribution rate in 15 ranges, with the highest being 59% in the Wuling Mts. Contribution rates from terrain relief and aspect are minimal, never exceeding 12%.

**FIGURE 4:**
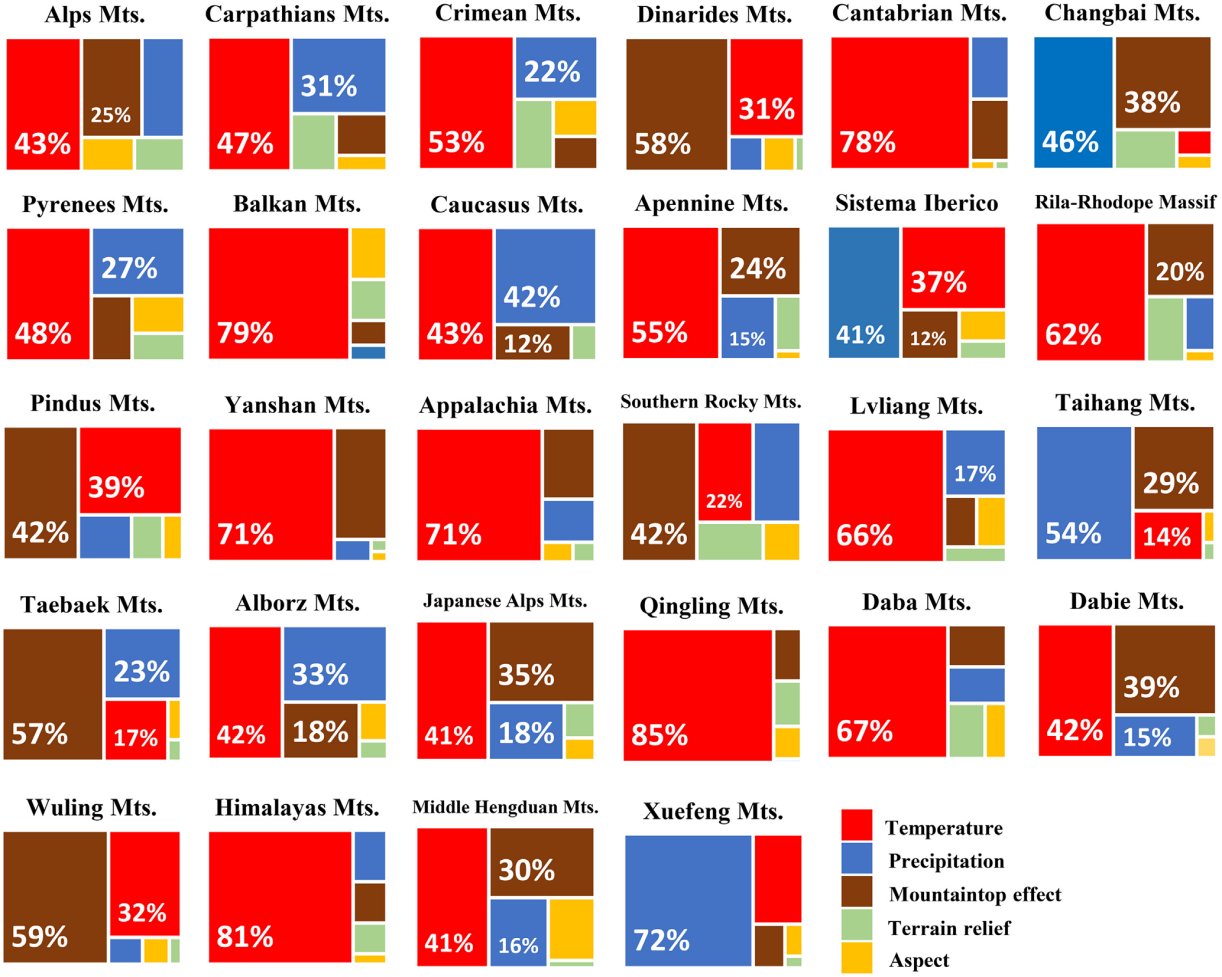
Contribution rates of various impact factors to ULMDBs. The white percentage labels indicate the contribution rate of each impact factor; factors with lower contributions are not labeled.

Temperature has a maximum contribution rate of 85% in the Qinling Mts., while precipitation reaches up to 72% in the Xuefeng Mts. Climate factors (combined temperature and precipitation) contribute over 50% to ULMDB distribution height in 21 mountains, with all ranges showing climate factor contribution rates exceeding 35%. Thus, climate factors exert a decisive influence on ULMDB distribution height in the Northern Hemisphere (Figure 4).

### 3.3 Regional Differences in ULMDB Sensitivity to Climate Change

The temperature sensitivity index (TSI), precipitation sensitivity index (PSI), and comprehensive sensitivity index (CSI) for ULMDB sensitivity to climate change are presented in (Table 2). For temperature sensitivity, in humid regions, 70% of the mountains have a TSI contribution rate exceeding 50%, indicating higher sensitivity to temperature changes. Among these, the Daba Mts. show the highest TSI (10.602), while the Xuefeng Mts. have the lowest (1.237). In arid and semi‐arid regions, the Yanshan Mts. exhibit the highest TSI (4.637), and the Alborz Mts. the lowest (2.395).

**TABLE 2:** Sensitivity index of ULMDBs to climate change.

Mountains	Latitude	Longitude	Sensitivity index			Contribution rate	
			Temperature	Precipitation	Comprehensive	Temperature	Precipitation
Carpathians Mts.	46.1° N	24.0° E	3.955	2.561	6.516	60.70%	39.30%
Alps Mts.	46.2° N	10.6° E	1.967	1.389	3.356	58.62%	41.38%
Crimean Mts.	44.6° N	34.2° E	1.662	1.016	2.678	52.05%	37.95%
Dinarides Mts.	43.6° N	18.2° E	3.453	2.031	5.483	62.97%	37.03%
Changbai Mts.	42.8° N	127.2° E	5.857	5.303	11.161	52.48%	47.52%
Caucasus Mts.	42.7° N	42.9° E	3.891	3.713	7.604	51.17%	48.83%
Balkan Mts.	42.7° N	24.2° E	4.381	4.243	8.625	50.80%	49.20%
Apennine Mts.	42.3° N	13.1° E	5.764	7.763	13.527	42.61%	57.39%
Rila‐Rhodope Massif	41.8° N	23.0° E	3.798	11.484	15.282	24.85%	75.15%
Pindus Mts.	40.7° N	20.4° E	2.466	4.379	6.845	36.02%	63.98%
Yanshan Mts.*	40.3° N	116.7° E	4.637	8.843	13.480	34.40%	65.60%
Appalachia Mts.	39.7° N	78.16° W	2.441	5.475	7.916	30.84%	69.16%
Southern Rocky Mts.*	39.0° N	105.8° W	4.107	4.498	8.605	47.73%	52.27%
Lvliang Mts.*	37.9° N	111.5° E	3.157	6.197	9.354	33.75%	66.25%
Taihang Mts.*	37.8° N	113.8° E	3.669	6.817	10.487	34.99%	65.01%
Taebaek Mts.	37.7° N	128.6° E	5.153	3.536	8.689	59.31%	40.69%
Alborz Mts.*	37.1° N	50.6° E	2.395	1.763	4.158	57.60%	42.40%
Japanese Alps Mts.	36.2° N	138.0° E	1.561	3.437	4.998	31.24%	68.76%
Qingling Mts.	33.7° N	108.8° E	8.178	6.971	15.150	53.98%	46.02%
Daba Mts.	31.9° N	108.8° E	10.603	4.618	15.221	69.66%	30.34%
Dabie Mts.	31.4° N	115.9° E	3.766	1.979	5.744	65.56%	34.44%
Cantabrian Mts.	43.1° N	4.0° W	2.473	1.394	3.867	63.95%	36.05%
Pyrenees Mts.	42.8° N	0.2° W	3.169	3.350	6.519	48.61%	51.39%
Himalayas Mts.	29.0° N	83.0° E	3.110	2.767	5.877	52.92%	47.08%
Middle Hengduan Mts.	27.6° N	99.6° E	3.702	2.805	6.507	56.90%	43.10%
Sistema Iberico	42.1° N	2.8° W	9.640	2.692	12.332	78.17%	21.83%
Wuling Mts.	29.4° N	109.2° E	3.257	9.426	12.683	25.68%	74.32%
Xuefeng Mts.	26.7° N	110.7° E	1.237	0.797	2.034	60.81%	39.19%

*Note:* Gray represents subarctic climate; blue represents humid continental climate; red represents oceanic climate; yellow represents mediterranean climate; and green represents humid subtropical climate. Mountains marked with an * symbol represent arid and semi‐arid regions. To facilitate subsequent data analysis, all sensitivity indices are multiplied by 100.

For precipitation sensitivity, in arid and semi‐arid regions, 80% of the mountains have a PSI contribution rate exceeding 50%, indicating high sensitivity to precipitation changes. In humid regions, the Rila‐Rhodope Massif has the highest PSI (11.484), and the Xuefeng Mts. has the lowest (0.797). In arid and semi‐arid regions, the Yanshan Mts. show the highest PSI (8.843), and the Alborz Mts. has the lowest (1.763).

The sum of TSI and PSI was defined as the total sensitivity to climate change, and the contribution rates of temperature and precipitation sensitivities were calculated to compare differences across mountains. For TSI contribution rate, the Sistema Iberico in humid regions has the highest rate (78.17%), while the Alborz Mts. in arid and semi‐arid regions have the highest (57.6%). For PSI contribution rate, the Rila‐Rhodope Massif in humid regions has the highest rate (75.15%), and the Lvliang Mts. in arid and semi‐arid regions have the highest (66.25%).

In humid continental climate zones, 58% of mountains have a TSI contribution rate exceeding 50%, indicating greater sensitivity to temperature than precipitation. The limited number of ULMDB locations in other climate zones restricts the statistical reliability of sensitivity analyses.

The mean CSI in the East Asian study region (9.909) is higher than in the European study region (7.730) and other Northern Hemisphere regions (6.778). East Asian study region's pronounced monsoon climate, high plant diversity, species richness, widespread distribution of deciduous broad‐leaved forests, complex topography, and significant climate gradients (Wang et al. 2021) collectively contribute to the high climate change sensitivity of ULMDBs in this region.

**FIGURE 5:**
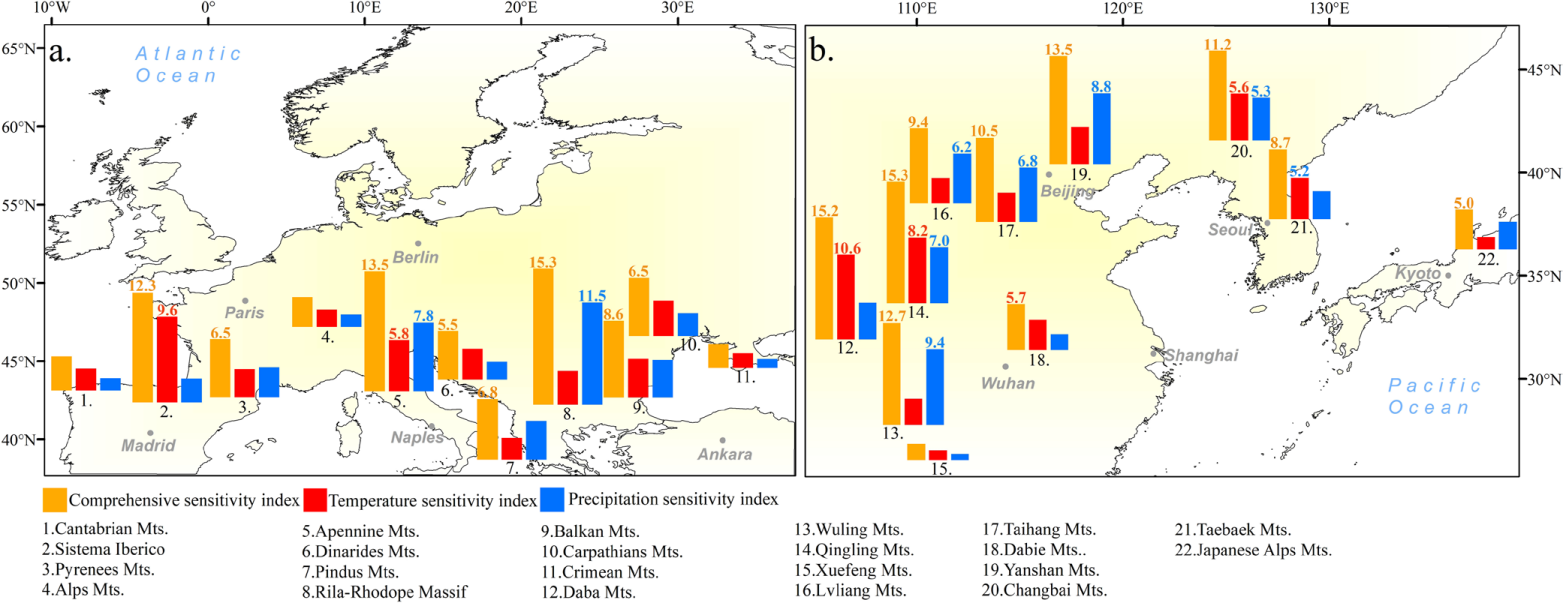
Regional differences in sensitivity indices of ULMDBs across mountains in Europe and East Asia. (a, b) represent mountains in Europe and East Asia, respectively. Mountain locations were determined by the mean latitude and longitude of their corresponding ULMDB points. To avoid overlap, the positions of some bars have been slightly adjusted.

Regional differences in the sensitivity indices of ULMDBs in European and East Asian study regions (Figure 5) show that in the European study region, CSI decreases with increasing latitude. The highest CSI (15.282) occurs in the lower‐latitude Rila‐Rhodope Massif (41.8° N). Higher CSI values in the European study region (Sistema Iberico, Apennine Mts., and Rila‐Rhodope Massif) are concentrated near Mediterranean peninsulas, increasing from west to east (12.332, 13.527, 15.282). For single‐factor sensitivity, the maximum TSI (9.640) occurs in the Sistema Iberico (42.1° N), and the maximum PSI (11.484) occurs in the Rila‐Rhodope Massif (41.8° N).

In East Asian study region, CSI exhibits a rise‐then‐fall trend with latitude, peaking at 15.221 in the Daba Mts. (31.9° N). CSI generally increases from coastal to inland areas, showing a northwest‐high, southeast‐low pattern. Mountains with high CSI, such as the Daba Mts. (15.221), Qinling Mts. (15.150), and Yanshan Mts. (13.480), are typically located in arid and semi‐arid regions or near the boundary between humid and arid zones. For single‐factor sensitivity, the maximum TSI (10.602) occurs in the Daba Mts. (31.9° N), and the maximum PSI (9.426) in the Yanshan Mts. (40.3° N).

**FIGURE 6:**
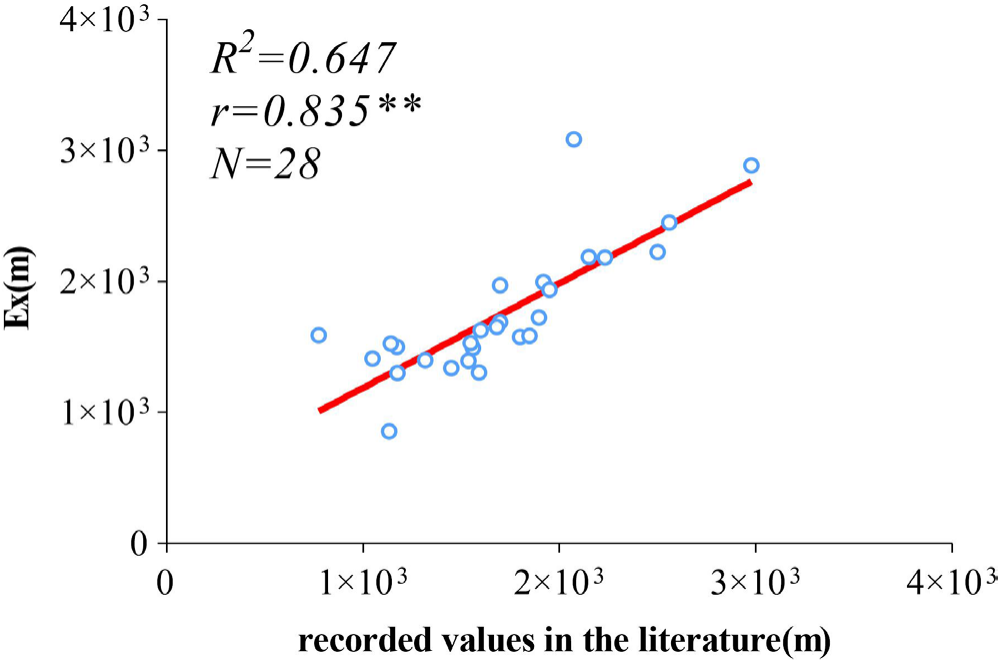
Correlation analysis between Ex values of ULMDB distribution height and literature records for 28 mountains in the Northern Hemisphere. r denotes the correlation coefficient; ** indicates significance at ρ < 0.01; R^2^ represents the coefficient of determination; N is the sample size; the red line indicates the fitted regression. Literature values for each mountain were derived from the average of reported values within each analysis unit.

Correlation analysis between the Ex of ULMDB distribution height and literature‐recorded values (Figure 6) shows a correlation coefficient of 0.911 (ρ < 0.001) and an *R* ^*2*^ of 0.750 for the fitted line, indicating that combining high‐resolution land cover data and Google Earth imagery can accurately extract ULMDB distribution height.

Validation results for the sensitivity indices (Table 3) demonstrate significant correlations between TSI, PSI, and CSI and the corresponding NDVI change rates across mountains, effectively explaining ULMDB responses to climate change.

**TABLE 3:** Verification of climate change sensitivity index for ULMDBs.

Sensitivity index	${\text{NDVI}}_{\text{slope}}$
Temperature	0.432*
Precipitation	0.588**
Comprehensive	0.556**

** Correlation is significant at the 0.01 level (two‐tailed).

* Correlation is significant at the 0.05 level (two‐tailed).

## 4 Discussion

### 4.1 Climate Indicators and Data Considerations

Temperature has long been recognized as the primary determinant of timberline elevation (Jiao et al. 2018), yet its role varies substantially among regions (Zhao et al. 2018), even within a single mountain system (Körner 1998; Troll 1973). Global timberline patterns are jointly shaped by thermal constraints and regional modifiers (Körner 1998), but there remains no consensus on the selection of temperature metrics. Considering the diverse climatic settings across our study domain, we used the mean January temperature as an indicator of species cold tolerance. This metric has been widely applied in monsoon‐dominated regions of eastern China, as well as in Mediterranean and sub‐boreal oceanic climates (Zhao et al. 2018; Wang et al. 2024).

To ensure well‐developed altitudinal vegetation zonation (Li, Yao, et al. 2023), we only included ULMDB data above 700 m a.s.l. In arid interior regions such as western North America and central–western Asia, deciduous broad‐leaved forests are limited by water and energy availability, typically occurring on moist slopes (Peet 1978) or in highly fragmented patches (Tan 2009). Together with ecosystem fragility and anthropogenic disturbances, these conditions pose challenges for remote sensing detection. Future work could integrate machine learning approaches (Lin et al. 2024; Shan et al. 2023; Zhang 2023) to improve ULMDB mapping accuracy in drylands.

Species‐specific climate sensitivities are also evident. For instance, *Fagus sylvatica* is highly sensitive to drought (del Río et al. 2021; Dulamsuren et al. 2017; Knutzen et al. 2015), whereas *Quercus ilex* exhibits greater drought tolerance than Q. pyrenaica (Gea‐Izquierdo et al. 2013). The distribution of Betula species is largely governed by seasonal temperature variability (Huang et al. 2025). Previous studies have shown (Holtmeier 2003a) that species' tolerance to extreme environments strongly influences their sensitivity to climate change. Due to limitations in remote sensing resolution and data availability, species‐level differences were not explicitly incorporated into our modeling framework. Integrating species composition in future work would likely enhance the precision of regional sensitivity assessments.

### 4.2 Regional Differences in ULMDB Sensitivity

This study's analysis reveals significant regional differences in ULMDB sensitivity to climate factors across the Northern Hemisphere. ULMDBs in humid regions are primarily driven by temperature changes, while in arid and semi‐arid regions, they are more sensitive to precipitation changes. Conventionally, timberline formation is attributed to thermal imbalances caused by increasing elevation (Heikkinen et al. 2002; Wang et al. 2004). While insufficient precipitation significantly weakens tree vigor, affects vegetation regeneration, and limits growth (Du et al. 2014; Park and Sohn 2010; Zhang et al. 2016), the influence of temperature remains substantial. In arid and semi‐arid regions, the mechanisms underlying the influence of temperature on ULMDBs lack a unified explanation, encompassing direct effects on the growing season (Lu et al. 2019) and carbon balance (Shi 1999), modulated by local geographical conditions. although the Alborz Mountains in northern Iran are located within an arid and semi‐arid region, their proximity to the Caspian Sea provides relatively favorable moisture conditions, partially alleviating water limitations. As a result, ULMDBs in this region exhibit a relatively strong temperature‐dominated pattern, with a TSI contribution rate of 57.60% (Ballato et al. 2010). Conversely, while humid regions have ample precipitation, some ULMDB locations still exhibit high sensitivity to precipitation changes. The Japanese Alps Mts. on Honshu Island, with a PSI contribution rate of 69.16%, are influenced by complex island climate factors such as winter snow accumulation (Tan 2009; Karger et al. 2019; Tanaka 1998; Yoshino 1978). Globally, most alpine timberlines are primarily temperature‐limited, but in arid regions, moisture significantly influences timberline elevation (Holtmeier 2003a; Körner 2012). Integrating previous studies and this study's findings, ULMDBs in arid and semi‐arid regions are generally more sensitive to precipitation changes, while in humid regions, it is more sensitive to temperature changes—a pattern with broad applicability.

### 4.3 Regional Differences in Timberline Dynamics

Over the long term, sustained global warming will drive timberlines to higher elevations and latitudes (Holtmeier and Broll 2005). However, response mechanisms are strongly modulated by regional climate backgrounds and local ecological conditions (Holtmeier 2003b). For example, in the Alps, timberlines dominated by *Pinus cembra* tend to remain relatively stable under maritime climatic conditions, whereas in continental climates, the same species shows upward shifts in response to increasing temperatures due to its strong drought tolerance (Compostella and Caccianiga 2017). In the Himalayas, arid timberlines are more likely to retreat (Lyu et al. 2019), whereas upward shifts in humid regions remain temperature‐driven. These differences highlight the significant influence of regional climate types on timberline dynamics (Troll 1973). While traditional views emphasize low‐temperature constraints on timberline distribution (Körner 2012; Körner and Paulsen 2004), recent studies suggest that the complexity of drought stress on timberline species far exceeds that of low‐temperature stress, with severe drought potentially negating the positive effects of warming (Panthi et al. 2020; Pandey et al. 2025; Teskey et al. 2015). On the basis of the sensitivity index system constructed in this study, incorporating temperature, precipitation, and comprehensive sensitivities, and considering the intensity of climate change over the past two decades, we suggest that timberlines with high TSI (e.g., Daba Mts., Qinling Mts.) have the greatest potential for upward migration, followed by those with high CSI (e.g., Rila‐Rhodope Massif, Apennine Mts.), while timberlines with high PSI (e.g., Wuling Mts., Appalachian Mts.) tend to exhibit relatively lower upward migration potential. However, timberline location changes are influenced by multiple factors. Of the 166 globally reported timberline sites, only about 52% exhibited significant upward shifts over the past century (Harsch et al. 2009), with some changes closely linked to human disturbances (Liang et al. 2011; Wang and Liang 2012). Compared to timberline location, stand density may be more sensitive to climate change (Camarero and Gutiérrez 2004; Elliott and Baker 2004; Wang and Liang 2012). Thus, the three sensitivity indices proposed in this study may better reflect changes in timberline stand density in the study area.

It should be noted that timberlines are a phenomenon related to space and time, and the spatial heterogeneity of impact factors may also stem from historical and anthropogenic causes (Holtmeier and Broll 2005). The heterogeneity of factors influencing timberline location increases as scale decreases, and the key factors determining timberline distribution may differ across scales, with the specific mechanisms driving this spatial heterogeneity remaining unclear (Lyu et al. 2019; Turner 1961). This study provides a method and possibility for comparing and discussing timberline responses to climate change across mountains at a large scale. It should be emphasized that these results represent potential response directions inferred from sensitivity analysis, rather than direct evidence of actual shifts in timberline position.

### 4.4 Mechanisms and Implications of Regional Differences

The pronounced regional differences in ULMDB sensitivity to climate change reveal deeper insights into the spatial complexity of ecosystem–climate interactions.

In addition to climatic factors, geomorphological conditions play a critical role in regulating ULMDB sensitivity by modulating regional hydrothermal conditions and local microclimates. For example, slope aspect and terrain relief influence solar radiation and soil moisture redistribution, thereby generating substantial microclimatic variability within the same mountain system and affecting the response intensity of montane forests to temperature and precipitation changes. At the local scale, such geomorphological effects may exert influences comparable to those of climatic factors (Holtmeier and Broll 2005). At the regional scale, large mountain systems reshape precipitation patterns through orographic uplift and airflow blocking, resulting in clear windward and leeward contrasts. In this study, although the Pyrenees in northern Spain are located in a humid region, the mountain barrier limits moisture transport from the Atlantic Ocean, creating a typical rain shadow on the southern slope. As a result, ULMDBs in this region exhibit a precipitation‐dominated characteristic, with a PSI contribution rate of 51.39%.

Moreover, differences in geomorphological characteristics among mountain systems can also lead to significant variations in ULMDB sensitivity. For instance, the Daba Mountains and the Dabie Mountains are located at similar latitudes and share comparable climate types, yet their sensitivity indices differ markedly. The Daba Mountains are characterized by a larger spatial extent, stronger terrain relief, and higher landscape fragmentation, which lead to greater spatial heterogeneity in hydrothermal conditions. In contrast, the Dabie Mountains are smaller in scale, with relatively gentle terrain and more homogeneous hydrothermal conditions. These differences indicate that geomorphology is an important factor contributing to the divergence in ULMDB sensitivity between the two regions.

It should be noted that although this study quantitatively analyzed factors such as aspect, slope, and terrain relief, their digital features did not show significant correlations with ULMDB sensitivity indices, as presented in Table 4. This suggests that the influence of geomorphology is nonlinear and region‐dependent, making it difficult to represent using a single quantitative indicator at large spatial scales. Therefore, geomorphological factors are more suitable for mechanism interpretation rather than as unified explanatory variables.

**TABLE 4:** Correlation analysis between geomorphological factors and sensitivity indices. *r* represents the correlation coefficient, and *p* denotes the significance level.

En		Sensitivity index		
		Temperature	Precipitation	Comprehensive
Aspect	*r*	0.500	−0.105	−0.026
	*p*	0.801	0.597	0.879
Terrain relief	*r*	−0.159	−0.138	−0.149
	*p*	0.418	0.484	0.448
Mountaintop effect	*r*	0.014	−0.079	−0.064
	*p*	0.945	0.690	0.746

Under contrasting moisture regimes, humid regions experience abundant and relatively stable precipitation, ensuring sufficient water supply during the growing season. Here, temperature becomes the principal limiting factor, influencing key physiological processes such as photosynthesis (Hikosaka et al. 2006; Mathur et al. 2014), transpiration (Gates 1968; Sadok et al. 2021), and phenology. In contrast, arid and semi‐arid regions face chronic soil moisture deficits. Trees often rely on deep rooting systems, water storage, or snowmelt to sustain physiological activity (Cleverly et al. 2016; Grayson et al. 2006; O'Donnell and Manier 2022). Consequently, growth is strongly dependent on the timing and variability of precipitation, rendering water availability the dominant constraint. Yet, even where ULMDBs exhibit sensitivity to the same climate variable, ecosystem responses remain highly idiosyncratic. Both temperature‐sensitive and precipitation‐sensitive ULMDBs demonstrate unique adaptive behaviors rather than conforming to a uniform rule. For example, the progressive aridification of the Caucasus (Nikolaishvili and Matchavariani 2015; Shatberashvili et al. 2015) and the upward migration of vegetation belts in the Apennines (Rogora et al. 2018) indicate that temperature, precipitation, regional ecological processes, species adaptation, and human activities jointly shape these complex responses. Ecosystem changes therefore cannot be attributed to a single climatic factor. Understanding these mechanisms is essential for predicting the long‐term impacts of climate change on montane forests.

At broader spatial scales, these regional differences are particularly pronounced across the Eurasian continent. In East Asian study region, ULMDBs spans a broad latitudinal range (26° N–42° N), under monsoon‐dominated climates with pronounced interannual hydrothermal variability, high species diversity, and complex orography, resulting in strongly developed vertical ecosystem structure (Wang et al. 2021). Notably, the Qinling–Daba Mountain range, located at the transition between subtropical and temperate zones, exhibits a pronounced peak in climate change sensitivity within mid‐latitude regions characterized by moderate hydrothermal conditions (Zhang 2019; Zhang et al. 2022). By contrast, ULMDBs in European study region is mainly confined to 42° N–46° N, at higher overall latitudes, under maritime climatic moderation with smaller thermal amplitudes. Combined with lower deciduous species diversity (Barnes 1991), simpler ecosystem structure, and north–south contrasts in climate change impacts (Bredemeier 2011; Ciscar et al. 2018), this region shows a decreasing trend in climate change sensitivity with increasing latitude. These differences not only reflect shifts in dominant climatic drivers but also capture the long‐term interplay of species assemblages, topographic configurations, and climatic regimes in shaping regional ecological characteristics.

## 5 Conclusions

This study develops and validates a cloud model–based framework to quantify ULMDB sensitivity to climate change at the hemispheric scale, enabling a robust and unified assessment of temperature, precipitation, and comprehensive sensitivity under uncertainty.

ULMDB sensitivity exhibits a clear dry and wet contrast, with temperature dominance in humid regions and precipitation control in arid and semi‐arid regions, reflecting a shift in dominant climatic drivers. Sensitivity also shows strong spatial heterogeneity, shaped by continental‐scale climate patterns and regional environmental conditions.

Overall, the proposed framework provides an effective quantitative approach for assessing the climate sensitivity of montane forest ecotones and has potential for application to other climate‐sensitive ecological boundaries.

## Author Contributions

**Youheng Li:** data curation (equal), methodology (equal), software (equal), writing – original draft (equal), writing – review and editing (equal). **Fang Han:** conceptualization (equal), methodology (equal), project administration (equal), writing – review and editing (equal). **Chuanrong Li:** conceptualization (equal), funding acquisition (equal), project administration (equal). **Kun Li:** investigation (equal), resources (equal). **Xiaoyong Li:** supervision (equal). **Yan Lv:** supervision (equal). **Xiaolong Xu:** validation (equal). **Junxin Zhao:** validation (equal). **Ziqiang Lei:** software (equal).

## Funding

This work was supported by the National Natural Science Foundation of China (41401111), Open Research Fund Program of Shandong Provincial Key Laboratory of Eco‐Environmental Science for Yellow River Delta (2025KFJJ04).

## Conflicts of Interest

The authors declare no conflicts of interest.

## Supporting information


**Data S1:** ece373561‐sup‐0001‐Supinfo.xlsx.

## Data Availability

Data and code are available at https://figshare.com/s/fc9d84dc8006c7af8fc1

## References

[ece373561-bib-0001] Bakker, M. R. , I. Brunner , F. Ashwood , et al. 2019. “Belowground Biodiversity Relates Positively to Ecosystem Services of European Forests.” Frontiers in Forests and Global Change 2, no. 6: 1–14. 10.3389/ffgc.2019.00006.

[ece373561-bib-0002] Ballato, P. , A. Mulch , A. Landgraf , et al. 2010. “Middle to Late Miocene Middle Eastern Climate From Stable Oxygen and Carbon Isotope Data, Southern Alborz Mountains, N Iran.” Earth and Planetary Science Letters 300, no. 1–2: 125–138. 10.1016/j.epsl.2010.09.043.

[ece373561-bib-0003] Barnes, B. V. 1991. “Deciduous Forests of North America.” In Ecosystems of the World, edited by D. W. Goodall , 219–344. Elsevier.

[ece373561-bib-0004] Beck, H. E. , T. R. McVicar , N. Vergopolan , et al. 2023. High‐Resolution (1 km) Köppen‐Geiger Maps for 1901–2099 Based on Constrained CMIP6 Projections. *Figshare*, v2. [Dataset]. 10.6084/m9.figshare.21789074. PMC10593765 37872197

[ece373561-bib-0005] Bonanomi, G. , M. Zotti , V. Mogavero , et al. 2020. “Climatic and Anthropogenic Factors Explain the Variability of *Fagus sylvatica* Treeline Elevation in Fifteen Mountain Groups Across the Apennines.” Forest Ecosystems 7, no. 1: 5. 10.1186/s40663-020-0217-8.

[ece373561-bib-0006] Bredemeier, M. 2011. “Forest Management and the Water Cycle–Introduction to the Challenge.” In An Ecosystem‐Based Approach, edited by M. Bredemeier , S. Cohen , D. L. Godbold , E. Lode , V. Pichler , and P. Schleppi , ix–xv. Springer.

[ece373561-bib-0007] Cairns, D. M. 2013. “Review of “Alpine Treelines: Functional Ecology of the Global High Elevation Tree Limits”.” Arctic, Antarctic, and Alpine Research 45, no. 3: 420–421. 10.1657/1938-4246-45.3.420.

[ece373561-bib-0008] Camarero, J. J. , and E. Gutiérrez . 2004. “Pace and Pattern of Recent Treeline Dynamics: Response of Ecotones to Climatic Variability in the Spanish Pyrenees.” Climatic Change 63, no. 1: 181–200. 10.1023/B:CLIM.0000018507.71343.46.

[ece373561-bib-0009] Chen, C. , J. Y. Liao , Y. Liu , et al. 2025. “Predicting Forest Carbon Sequestration of Ecological Buffer Zone in Urban Agglomeration: Integrating Vertical Heterogeneity and Age Class Dynamics to Unveil Future Trajectories.” Forests 16, no. 11: 1648. 10.3390/f16111648.

[ece373561-bib-0010] Chen, X. X. , Q. R. Chang , B. Y. Guo , and X. J. Zhang . 2013. “Analytical Study of the Relief Amplitude in China Based on SRTM DEM Data.” Journal of Basic Science and Engineering 21, no. 4: 670–678. 10.3969/j.issn.1005-0930.2013.04.009.

[ece373561-bib-0011] Ciscar, J. C. , D. Ibarreta , A. Soria , et al. 2018. Climate Impacts in Europe: Final Report of the JRC PESETA III Project, EUR 29427 EN. Publications Office of the European Union. 10.2760/93257.

[ece373561-bib-0012] Cleverly, J. , D. Eamus , N. R. Coupe , et al. 2016. “Soil Moisture Controls on Phenology and Productivity in a Semi‐Arid Critical Zone.” Science of the Total Environment 568: 1227–1237. 10.1016/j.scitotenv.2016.05.142. 27241203

[ece373561-bib-0013] Cogbill, C. V. , and P. S. White . 1991. “The Latitude–Elevation Relationship for Spruce‐Fir Forest and Treeline Along the Appalachian Mountain Chain.” Vegetatio 94, no. 2: 153–175. 10.1007/BF00032629.

[ece373561-bib-0014] Compostella, C. , and M. Caccianiga . 2017. “A Comparison Between Different Treeline Types Shows Contrasting Responses to Climate Fluctuations.” Plant Biosystems 151, no. 3: 436–449. 10.1080/11263504.2016.1179695.

[ece373561-bib-0016] del Río, S. , R. Canas , E. Cano , et al. 2021. “Modelling the Impacts of Climate Change on Habitat Suitability and Vulnerability in Deciduous Forests in Spain.” Ecological Indicators 131: 108202. 10.1016/j.ecolind.2021.108202.

[ece373561-bib-0017] Dhar, U. 2012. “Himalayan Ecology and Environment: Redefining Paradigms.” Journal of Tropical Ecology 53, no. 3: 241–244.

[ece373561-bib-0018] Didan, K. 2021. “MODIS/Terra Vegetation Indices 16‐Day L3 Global 1km SIN Grid V061. NASA Land Processes Distributed Active Archive Center (LP DAAC), v061.” [dataset]. 10.5067/MODIS/MOD13A2.061.

[ece373561-bib-0019] Dong, R. , N. Li , M. H. Li , Y. Cong , H. Du , and H. S. He . 2025. “Treeline Species Betula Ermanii Are More Adaptable to Alpine Environments Than Non‐Treeline Species Picea Jezoensis: Evidence From Leaf Functional Traits.” EGUsphere [Preprint]. 10.5194/egusphere-2025-369.

[ece373561-bib-0020] Du, J. , Z. B. He , J. J. Yang , L. F. Chen , and X. Zhu . 2014. “Detecting the Effects of Climate Change on Canopy Phenology in Coniferous Forests in Semi‐Arid Mountain Regions of China.” International Journal of Remote Sensing 35, no. 17: 6490–6507. 10.1080/01431161.2014.955146.

[ece373561-bib-0021] Dulamsuren, C. , M. Hauck , G. Kopp , M. Ruff , and C. Leuschner . 2017. “European Beech Responds to Climate Change With Growth Decline at Lower, and Growth Increase at Higher Elevations in the Center of Its Distribution Range (SW Germany).” Trees 31: 673–686. 10.1007/s00468-016-1499-x.

[ece373561-bib-0022] Elliott, G. P. , and W. L. Baker . 2004. “Quaking Aspen ( *Populus tremuloides* Michx.) at Treeline: A Century of Change in the San Juan Mountains, Colorado, USA.” Journal of Biogeography 31, no. 5: 733–745. 10.1111/j.1365-2699.2004.01064.x.

[ece373561-bib-0023] Fang, J. Y. , S. L. Piao , L. M. Zhou , et al. 2005. “Precipitation Patterns Alter Growth of Temperate Vegetation.” Geophysical Research Letters 32, no. 21: L21411. 10.1029/2005GL024231.

[ece373561-bib-0024] Fankhauser, F. 1901. “Der Oberste Baumwuchs.” Schweiz. Z. ‐fFür Forstwes 2: 1–5.

[ece373561-bib-0025] Gao, W. Q. , X. D. Lei , M. W. Liang , et al. 2021. “Biodiversity Increased Both Productivity and Its Spatial Stability in Temperate Forests in Northeastern China.” Science of the Total Environment 780: 146674. 10.1016/j.scitotenv.2021.146674. 34030338

[ece373561-bib-0026] Gates, D. M. 1968. “Transpiration and Leaf Temperature.” Annual Review of Plant Biology 19, no. 1: 211–238. 10.1146/annurev.pp.19.060168.001235.

[ece373561-bib-0027] Gea‐Izquierdo, G. , L. Fernández‐de‐Uña , and I. Cañellas . 2013. “Growth Projections Reveal Local Vulnerability of Mediterranean Oaks With Rising Temperatures.” Forest Ecology and Management 305: 282–293. 10.1016/j.foreco.2013.05.058.

[ece373561-bib-0028] Gottfried, M. , H. Pauli , A. Futschik , et al. 2012. “Continent‐Wide Response of Mountain Vegetation to Climate Change.” Nature Climate Change 2: 111–115. 10.1038/nclimate1329.

[ece373561-bib-0029] Grayson, R. B. , A. W. Western , J. P. Walker , D. D. Kandel , J. F. Costelloe , and D. J. Wilson . 2006. “Controls on Patterns of Soil Moisture in Arid and Semi‐Arid Systems.” In Dryland Ecohydrology, edited by P. D'Odorico , A. Porporato , and C. Wilkinson Runyan , 109–127. Springer.

[ece373561-bib-0030] Griesbauer, H. , and A. Bevington . 2024. “Recent Changes in Forest Structure and Growth at the Alpine‐Treeline Ecotone in the Rocky and Columbia Mountains of Central British Columbia, Canada.” Journal of Biogeography 51, no. 4: 677–693. 10.1111/jbi.14778.

[ece373561-bib-0031] Harsch, M. A. , P. E. Hulme , M. S. McGlone , and R. P. Duncan . 2009. “Are Treelines Advancing? A Global Meta‐Analysis of Treeline Response to Climate Warming.” Ecology Letters 12, no. 10: 1040–1049. 10.1111/j.1461-0248.2009.01355.x. 19682007

[ece373561-bib-0032] He, W. H. , B. P. Zhang , Y. Pang , F. Zhao , W. W. Qi , and S. Zhang . 2015. “Effect of Slope Aspect on the Distribution of Mountain Forest in the Northern Flank of the Central Tianshan Mountains.” Mountain Research 33, no. 5: 546–552. 10.16089/j.cnki.1008-2786.000068.

[ece373561-bib-0033] Heikkinen, O. , M. Tuovinen , and J. Autio . 2002. “What Determines the Timberline?” Fennia 180, no. 1–2: 67–74.

[ece373561-bib-0034] Hikosaka, K. , K. Ishikawa , A. Borjigidai , O. Muller , and Y. Onoda . 2006. “Temperature Acclimation of Photosynthesis: Mechanisms Involved in the Changes in Temperature Dependence of Photosynthetic Rate.” Journal of Experimental Botany 57, no. 2: 291–302. 10.1093/jxb/erj049. 16364948

[ece373561-bib-0035] Hobi, M. L. , B. Commarmot , and H. Bugmann . 2015. “Pattern and Process in the Largest Primeval Beech Forest of Europe (Ukrainian Carpathians).” Journal of Vegetation Science 26, no. 2: 323–336. 10.1111/jvs.12234.

[ece373561-bib-0036] Holtmeier, F. K. 2003a. “Physiognomic and Ecological Differentiation of Mountain Timberline.” In Mountain Timberlines: Ecology, Patchiness, and Dynamics, edited by F. K. Holtmeier , 29–256. Springer.

[ece373561-bib-0037] Holtmeier, F. K. 2003b. “Timberline Fluctuations.” In Mountain Timberlines: Ecology, Patchiness, and Dynamics, edited by F. K. Holtmeier , 257–285. Springer.

[ece373561-bib-0038] Holtmeier, F. K. , and G. Broll . 2005. “Sensitivity and Response of Northern Hemisphere Altitudinal and Polar Treelines to Environmental Change at Landscape and Local Scales.” Global Ecology and Biogeography 14, no. 5: 395–410. 10.1111/j.1466-822X.2005.00168.x.

[ece373561-bib-0039] Huang, Z. L. , C. L. Fu , C. Y. Li , et al. 2025. “Distributional Responses of Five Betula (Betulaceae) Species to Future Climate Change in China.” Forests 16, no. 3: 400. 10.3390/f16030400.

[ece373561-bib-0040] IPCC . 2023. “Summary for Policymakers.” In Climate Change 2023: Synthesis Report, edited by H. Lee and J. Romero , 1–34. IPCC. 10.59327/IPCC/AR6-9789291691647.001.

[ece373561-bib-0041] Jahed, R. R. , M. R. Kavousi , M. E. Farashiani , et al. 2020. “A Comparison of the Formation Rates and Composition of Tree‐Related Microhabitats in Beech‐Dominated Primeval Carpathian and Hyrcanian Forests.” Forests 11, no. 2: 144. 10.3390/f11020144.

[ece373561-bib-0042] Jiao, K. W. , J. B. Gao , S. Wu , and W. J. Hou . 2018. “Research Progress on the Response Processes of Vegetation Activity to Climate Change.” Acta Ecologica Sinica 38, no. 6: 2229–2238. 10.5846/stxb201702240305.

[ece373561-bib-0043] Karger, D. N. , O. Conrad , J. Böhner , et al. 2017. “CHELSA High‐Resolution Land Surface Temperature and Precipitation.” Dryad Digital Repository, v1. [dataset]. 10.5061/dryad.kd1d4.

[ece373561-bib-0044] Karger, D. N. , M. Kessler , O. Conrad , et al. 2019. “Why Tree Lines Are Lower on Islands—Climatic and Biogeographic Effects Hold the Answer.” Global Ecology and Biogeography 28, no. 6: 839–850. 10.1111/geb.12897.

[ece373561-bib-0045] Knutzen, F. , I. C. Meier , and C. Leuschner . 2015. “Does Reduced Precipitation Trigger Physiological and Morphological Drought Adaptations in European Beech ( *Fagus sylvatica* L.)? Comparing Provenances Across a Precipitation Gradient.” Tree Physiology 35, no. 9: 949–963. 10.1093/treephys/tpv057. 26209617

[ece373561-bib-0046] Körner, C. 1998. “A Re‐Assessment of High Elevation Treeline Positions and Their Explanation.” Oecologia 115: 445–459. 10.1007/s004420050540. 28308263

[ece373561-bib-0047] Körner, C. 2007. “Climatic Treelines: Conventions, Global Patterns, Causes (Klimatische Baumgrenzen: Konventionen, Globale Muster, Ursachen).” Erdkunde 61, no. 4: 316–324. 10.3112/erdkunde.2007.04.02.

[ece373561-bib-0048] Körner, C. 2012. “Treelines Will Be Understood Once the Functional Difference Between a Tree and a Shrub Is.” Ambio 41, no. Suppl 3: 197–206. 10.1007/s13280-012-0313-2. 22864694 PMC3535059

[ece373561-bib-0049] Körner, C. , and J. Paulsen . 2004. “A World‐Wide Study of High Altitude Treeline Temperatures.” Journal of Biogeography 31, no. 5: 713–732. 10.1111/j.1365-2699.2003.01043.x.

[ece373561-bib-0050] Li, D. J. , and H. P. Tao . 1989. “An Approach to the Synthetical Model for Various Vertical Nature Zone and Its Application.” Exploration of Nature 8: 29–35.

[ece373561-bib-0051] Li, J. Y. , Y. Y. Yao , J. J. Liu , and B. P. Zhang . 2023. “Variation Analysis of the Typical Altitudinal Belt Width in the Qinling‐Daba Mountains.” Natural Protected Areas 3, no. 2: 12–25. 10.12335/2096-8981.2022080101.

[ece373561-bib-0052] Li, M. , J. J. Wu , R. P. Su , et al. 2025. “Genome Analyses Provide Insights Into Engelhardia's Adaptation to East Asia Summer Monsoon.” Plant Diversity 47, no. 5: 718–732. 10.1016/j.pld.2025.07.003. 41054613 PMC12496538

[ece373561-bib-0053] Li, W. Q. , R. D. Manzanedo , Y. Jiang , et al. 2023. “Reassessment of Growth‐Climate Relations Indicates the Potential for Decline Across Eurasian Boreal Larch Forests.” Nature Communications 14, no. 1: 3358. 10.1038/s41467-023-39057-5. PMC10250375 37291110

[ece373561-bib-0054] Liang, E. Y. , B. Liu , L. P. Zhu , and Z. Y. Yin . 2011. “A Short Note on Linkage of Climatic Records Between a River Valley and the Upper Timberline in the Sygera Mountains, Southeastern Tibetan Plateau.” Global and Planetary Change Publishes 77, no. 1–2: 97–102. 10.1016/j.gloplacha.2011.04.005.

[ece373561-bib-0055] Lin, C. , L. S. Yang , R. L. Zhou , T. X. Zhang , Y. L. Han , and Y. X. Wang . 2024. “Spatial Pattern and Environmental Driving Factors of Treeline Elevations in Yulong Snow Mountain, China.” Forests 15, no. 7: 1261. 10.3390/f15071261.

[ece373561-bib-0056] Liu, L. Y. , and X. Zhang . 2020. “Global 30 m Fine Land Cover Product in 2020.” Data Sharing and Service Portal. [dataset]. 10.5281/zenodo.4280923.

[ece373561-bib-0057] Liu, Y. L. , R. Q. Guo , and S. C. Sun . 2010. “Variations in the Vertical Vegetation Zonation of Subtropical Chinese Mountains: The Importance of Climatic Seasonality.” Acta Ecologica Sinica 30, no. 14: 3912–3922.

[ece373561-bib-0058] Lu, D. , X. J. Zhao , Z. Q. Liu , Z. T. Du , T. Wu , and H. Zhang . 2019. “Responses of Vegetation Growth to Temperature During 1982–2015 in Xinjiang, China.” Journal of Applied Ecology 30, no. 7: 2165–2170. 10.13287/j.1001-9332.201907.023. 31418218

[ece373561-bib-0059] Lyu, L. X. , Q. B. Zhang , M. G. Pellatt , U. Büntgen , M. H. Li , and P. Cherubini . 2019. “Drought Limitation on Tree Growth at the Northern Hemisphere's Highest Tree Line.” Dendrochronologia 53: 40–47. 10.1016/j.dendro.2018.11.006.

[ece373561-bib-0060] Mathur, S. , D. Agrawal , and A. Jajoo . 2014. “Photosynthesis: Response to High Temperature Stress.” Journal of Photochemistry and Photobiology, B: Biology 137: 116–126. 10.1016/j.jphotobiol.2014.01.010. 24796250

[ece373561-bib-0061] Meng, H. H. , Y. G. Song , G. X. Hu , et al. 2025. “Evolution of East Asian Subtropical Evergreen Broad‐Leaved Forests: When and How?” Journal of Systematics and Evolution 63, no. 5: 1045–1060. 10.1111/jse.70001.

[ece373561-bib-0062] Mkrtchian, A. , and D. Mueller . 2025. “Climatic Determinants of the Carpathian Treeline and Its Projected Upward Shifts in Response to Climate Change.” Climatic Change 178: 109. 10.1007/s10584-025-03947-y.

[ece373561-bib-0063] Mu, H. X. , F. Han , X. M. Tang , Z. Y. Wang , and Z. Wang . 2023. “Comparison and Analysis of Timberline and Treeline Distribution Height and Influencing Factors of Baima Snow Mountain and Bogda Mountain Based on Cloud Model.” Geographical Research 42, no. 7: 1941–1956. 10.11821/dlyj020221045.

[ece373561-bib-0064] NASA JPL . 2013. “NASA Shuttle Radar Topography Mission Global 1 Arc Second.” NASA Land Processes Distributed Active Archive Center, v3. [Dataset]. 10.5067/MEASURES/SRTM/SRTMGL1.003.

[ece373561-bib-0065] Nikolaishvili, D. , and L. Matchavariani . 2015. “Impacts of Climate Change on Georgia's Mountain Ecosystems.” In Climate Change Impacts on High‐Altitude Ecosystems, edited by M. Öztürk , K. R. Hakeem , I. Faridah‐Hanum , and R. Efe , 245–274. Springer International.

[ece373561-bib-0066] O'Donnell, M. S. , and D. J. Manier . 2022. “Spatial Estimates of Soil Moisture for Understanding Ecological Potential and Risk: A Case Study for Arid and Semi‐Arid Ecosystems.” Land 11, no. 10: 1856. 10.3390/land11101856.

[ece373561-bib-0067] Ogle, K. , and J. F. Reynolds . 2004. “Plant Responses to Precipitation in Desert Ecosystems: Integrating Functional Types, Pulses, Thresholds, and Delays.” Oecologia 141: 282–294. 10.1007/s00442-004-1507-5. 15007725

[ece373561-bib-0068] Pandey, K. P. , C. Wellstein , A. Bräuning , and D. R. Bhuju . 2025. “Climatic Influence on Growth Performance of Abies Spectabilis in the Himalayas.” Forests 16, no. 3: 473. 10.3390/f16030473.

[ece373561-bib-0069] Panthi, S. , Z. X. Fan , P. van der Sleen , and P. A. Zuidema . 2020. “Long‐Term Physiological and Growth Responses of Himalayan Fir to Environmental Change Are Mediated by Mean Climate.” Global Change Biology 26, no. 3: 1778–1794. 10.1111/gcb.14910. 31696994

[ece373561-bib-0070] Park, H. S. , and B. Sohn . 2010. “Recent Trends in Changes of Vegetation Over East Asia Coupled With Temperature and Rainfall Variations.” Journal of Geophysical Research‐Atmospheres 115, no. D14: D14102. 10.1029/2009JD012752.

[ece373561-bib-0071] Peet, R. K. 1978. “Latitudinal Variation in Southern Rocky Mountain Forests.” Journal of Biogeography 5, no. 3: 275–289. 10.2307/3038041.

[ece373561-bib-0072] Rogora, M. , L. Frate , M. Carranza , et al. 2018. “Assessment of Climate Change Effects on Mountain Ecosystems Through a Cross‐Site Analysis in the Alps and Apennines.” Science of the Total Environment 624: 1429–1442. 10.1016/j.scitotenv.2017.12.155. 29929254

[ece373561-bib-0073] Sadok, W. , J. R. Lopez , and K. P. Smith . 2021. “Transpiration Increases Under High‐Temperature Stress: Potential Mechanisms, Trade‐Offs and Prospects for Crop Resilience in a Warming World.” Plant, Cell & Environment 44, no. 7: 2102–2116. 10.1111/pce.13970. 33278035

[ece373561-bib-0074] Shan, W. , G. C. Xu , Y. Wang , L. S. Qiu , Y. Guo , and C. C. Zhang . 2023. “Response of Alpine Timberline to Permafrost Degradation on Changbai Mountain.” Sustainability 15, no. 24: 16768. 10.3390/su152416768.

[ece373561-bib-0075] Shatberashvili, N. , I. Rucevska , H. Jørstad , et al. 2015. Outlook on Climate Change Adaptation in the South Caucasus Mountains. United Nations Environment Programme, GRID‐Arendal and Sustainable Caucasus.

[ece373561-bib-0076] Shen, W. , L. Zhang , and T. Luo . 2017. “Advances in the Study of the Limitations of Seedling Recruitment for Alpine Timberline Forests.” Acta Ecologica Sinica 37, no. 9: 2858–2868.

[ece373561-bib-0077] Shi, P. L. 1999. Study on Vegetation Ecology of Subalpine Timberline Ecotone [dissertation]. Institute of Geographic Sciences and Natural Resources Research, Beijing.

[ece373561-bib-0078] Snethlage, M. A. , J. Geschke , E. M. Spehn , et al. 2022a. “GMBA Mountain Inventory.” GMBA‐EarthEnv, v2. [dataset]. 10.48601/earthenv-t9k2-1407.

[ece373561-bib-0079] Snethlage, M. A. , J. Geschke , E. M. Spehn , et al. 2022b. “A Hierarchical Inventory of the World's Mountains for Global Comparative Mountain Science.” Scientific Data 9: 149. 10.1038/s41597-022-01256-y. 35365674 PMC8975823

[ece373561-bib-0080] Sun, J. , and G. W. Cheng . 2014. “Mountain Altitudinal Belt: A Review.” Journal Ecology and Environment Sciences 23, no. 9: 1544–1550.

[ece373561-bib-0081] Tan, J. 2009. Digital integration and analysis of vertical belt spectra in Eurasian mountains [dissertation]. Institute of Geographic Sciences and Natural Resources Research, Beijing.

[ece373561-bib-0082] Tanaka, N. 1998. “Field Studies on the Effects of Global Warming on Mountain Vegetation in Japan.” Global Environmental Research 1: 71–74.

[ece373561-bib-0083] Teskey, R. , T. Wertin , I. Bauweraerts , M. Ameye , M. A. McGuire , and K. Steppe . 2015. “Responses of Tree Species to Heat Waves and Extreme Heat Events.” Plant, Cell & Environment 38, no. 9: 1699–1712. 10.1111/pce.12417. 25065257

[ece373561-bib-0084] Trabucco, A. , and R. Zomer . 2019. Global Aridity Index and Potential Evapotranspiration (ET0) Climate Database, v2 [dataset]. 10.6084/m9.figshare.7504448.v3. PMC9287331 35840601

[ece373561-bib-0085] Troll, C. 1973. “The Upper Timberlines in Different Climatic Zones.” Arctic, Antarctic, and Alpine Research 5, no. 3: A3–A18. 10.1080/00040851.1973.12003712.

[ece373561-bib-0086] Tse‐Ring, K. , E. Sharma , N. Chettri , and A. Shrestha . 2010. Climate Change Vulnerability of Mountain Ecosystems in the Eastern Himalayas. ICIMOD.

[ece373561-bib-0087] Turner, H. 1961. “Die Niederschlags‐und Schneeverhältnisse.” Mitteilungen der Forstlichen Bundesversuchsanstalt Mariabrunn 59: 265–315.

[ece373561-bib-0088] UNEP . 1997. Report of the Governing Council on the Work of Its 19th Session, 27 January–7 February 1997, 3–4 April 1997. United Nations Environment Programme.

[ece373561-bib-0089] Wang, J. , B. P. Zhang , and Y. Y. Yao . 2021. “The Spatial Pattern of the Upper Limit of Montane Deciduous Broad‐Leaved Forests and Its Geographical Interpretation in the East Monsoon Realm of China.” Forests 12, no. 9: 1225. 10.3390/f12091225.

[ece373561-bib-0090] Wang, J. , B. P. Zhang , and W. J. Zhang . 2017. “Quantitative Research of Mass Elevation Effect in Colorado Rocky Mountains.” Geographical Research 36, no. 8: 1467–1477. 10.11821/dlyj201708006.

[ece373561-bib-0091] Wang, X. P. , L. Zhang , and J. Y. Fang . 2004. “Geographical Differences in Alpine Timberline and Its Climatic Interpretation in China.” Acta Geographica Sinica 59, no. 6: 871–879. 10.11821/xb200406009.

[ece373561-bib-0092] Wang, Y. F. , and E. Y. Liang . 2012. “A Review on Progresses in Treeline Dynamics and Climate Change.” Journal of Earth Environment 3, no. 3: 855–861. 10.7515/JEE201203002.

[ece373561-bib-0093] Wang, Z. Y. , F. Han , C. R. Li , K. Li , H. X. Mu , and Z. Wang . 2024. “Distribution Characteristics and Geographical Interpretation of the Upper Limit of Montane Deciduous Broad‐Leaved Forests in the Eastern Monsoon Region of China.” Acta Geographica Sinica 79, no. 1: 240–258. 10.11821/dlxb202401015.

[ece373561-bib-0094] Wei, J. X. , Z. G. Jiang , L. S. Yang , et al. 2024. “Community Composition and Structure in a 25 Ha Mid‐Subtropical Mountain Deciduous Broad‐Leaved Forest Dynamics Plot in Shennongjia, Hubei, China.” Biodiversity Science 32, no. 3: 23338. 10.17520/biods.2023338.

[ece373561-bib-0095] Wieser, G. 2012. “Lessons From the Timberline Ecotone in the Central Tyrolean Alps: A Review.” Plant Ecology and Diversity 5, no. 1: 127–139. 10.1080/17550874.2010.498062.

[ece373561-bib-0096] Wolfe, J. A. 1979. Temperature Parameters of Humid to Mesic Forests of Eastern Asia and Relation to Forests of Other Regions of the Northern Hemisphere and Australasia. U.S. Government Printing Office. 10.3133/pp1106.

[ece373561-bib-0097] Xie, J. T. , and L. Z. Cheng . 1994. “Species Diversity Characteristics of Deciduous Forests in the Warm Temperate Zone of North China.” Acta Ecologica Sinica 14, no. 4: 337–344.

[ece373561-bib-0098] Yan, W. P. , Q. Wang , Y. L. Guo , Q. Hu , M. Yang , and Y. D. An . 2023. “Indicator of Climate Variability: Low Treeline Displacement in Arid Valleys of Mountain Areas.” China. Journal of Mountain Science 20, no. 11: 3250–3265. 10.1007/s11629-023-8392-z.

[ece373561-bib-0099] Yoshino, M. 1978. “Altitudinal Vegetation Belts of Japan With Special Reference to Climatic Conditions.” Arctic, Antarctic, and Alpine Research 10, no. 2: 449–456. 10.1080/00040851.1978.12003985.

[ece373561-bib-0100] Yu, J. , Q. J. Liu , Q. Q. Xu , C. W. Luo , H. M. Wang , and J. Q. Li . 2015. “Variation of Vegetation Index in Response to Climate Change on the Eastern Slope of Changbai Mountain, Northeast China.” Chinese Journal of Applied & Environmental Biology 21, no. 2: 323–332. 10.3724/SP.J.1145.2014.08012.

[ece373561-bib-0101] Zeng, X. , Z. G. Hu , A. P. Chen , et al. 2022. “The Global Decline in the Sensitivity of Vegetation Productivity to Precipitation From 2001 to 2018.” Global Change Biology 28, no. 22: 6823–6833. 10.1111/gcb.16403. 36054066

[ece373561-bib-0102] Zhang, B. P. 2019. “Ten Major Scientific Issues Concerning the Study of China's North‐South Transitional Zone.” Progress in Geography 38, no. 3: 305–311. 10.18306/dlkxjz.2019.03.001.

[ece373561-bib-0103] Zhang, B. P. , Y. H. Yao , F. Xiao , et al. 2022. “The Finding and Significance of the Super Altitudinal Belt of Montane Deciduous Broad‐Leaved Forests in Central Qinling Mountains.” Acta Geographica Sinica 77, no. 9: 2236–2248. 10.11821/dlxb202209007.

[ece373561-bib-0104] Zhang, Q. , X. L. Yuan , X. Chen , G. P. Luo , and L. H. Li . 2016. “Vegetation Change and Its Response to Climate Change in Central Asia From 1982 to 2012.” Chinese Journal of Plant Ecology 40, no. 1: 13–23. 10.17521/Cjpe.2015.0236.

[ece373561-bib-0105] Zhang, T. Q. 2023. Advancing Multi‐Dimensional Boreal Forest Mapping Through Satellite‐Based Analysis of Forest Cover, Forest Height, and Timberline [Dissertation]. Ohio State University.

[ece373561-bib-0106] Zhao, F. , L. Q. Zhu , B. P. Zhang , F. Han , Y. Y. Yao , and Y. P. Cao . 2018. “Temperature Differences of Timberlines in the Eurasian Continent.” Acta Ecologica Sinica 38, no. 1: 263–272. 10.5846/stxb201511062253.

